# The New Face of Autophagy in Chronic Lymphocytic Leukemia

**DOI:** 10.3390/cancers15215279

**Published:** 2023-11-03

**Authors:** Gelina S. Kopeina, Boris Zhivotovsky

**Affiliations:** 1 Engelhardt Institute of Molecular Biology, Russian Academy of Sciences, 119991 Moscow, Russia; 2 Faculty of Medicine, Lomonosov Moscow State University, 119991 Moscow, Russia; 3 Division of Toxicology, Institute of Environmental Medicine, Karolinska Institute, P.O. Box 210, 17177 Stockholm, Sweden

Chronic lymphocytic leukemia (CLL) mainly afflicts adults and accounts for 25% of all new leukemia cases. CLL progresses slower than other types of leukemia do, so many people do not suspect they are ill for a long time. The efficacy of targeted therapy for patients with CLL has improved in recent years. High levels of the antiapoptotic protein Bcl-2 in CLL has enabled its successful treatment with venetoclax, an oral BH3 mimetic that targets Bcl-2, which was first approved by the Food and Drug Administration (FDA) for CLL in 2015. This agent triggers apoptosis in CLL cells regardless of the p53 status via direct mitochondrial outer membrane permeabilization [1]. Moreover, a recent study revealed that the combined first-line treatment with venetoclax and obinutuzumab or ibrutinib increased the 3-year progression-free survival rate compared with that of chemoimmunotherapy [2]. This positive outcome has prompted the search for new effective combinations of venetoclax with other agents for CLL therapy.

There are some limitations to venetoclax application that are mainly related to initial and acquired drug resistance. In some patients, this target therapy leads to a progressive disease associated with Richter’s transformation [3]. The mechanisms underlying insensitivity include factors such as an increased level of other antiapoptotic Bcl-2 family proteins (e.g., Mcl-1 or Bcl-XL), changes in metabolism, and the modulation of the AMPK–PKA axis [4,5]. 

Avsec et al. focused on the mechanisms of the autophagy-dependent resistance of CLL cells derived from patients and the ability of autophagic inhibitors to trigger programmed cell death (PCD) [6]. These authors demonstrated that three different autophagy-blocking agents with various mechanisms of action (dorsomorphin, MRT68921, and chloroquine) decreased the viability of CLL cells. The addition of a pan-caspase inhibitor could rescue the cells from the cytotoxic action of two of these three compounds; these data confirmed apoptosis induction upon autophagy inhibition. The link between these types of PCD is well known and can be observed in various normal and cancer cells [7]. However, the comparison of peripheral blood mononuclear cells (PBMCs) from healthy donors and patients with CLL revealed the selective action of autophagic inhibitors in the context of CLL. Previously, endoplasmic reticulum (ER) stress was found to induce autophagy in CLL cells, and its inhibition triggered apoptosis [8]. Interestingly, the first evidence of a similar effect was reported more than 20 years ago. The treatment of cells derived from patients with CLL with hydroxychloroquine increased the activity of caspase-3 and the proteolytic cleavage of poly(ADP-ribose) polymerase (PARP), and altered the Bcl-2/Bax ratio [9]. These studies have confirmed that autophagy has a prosurvival function for CLL cells, while the blockade of autophagy promotes apoptosis. Importantly, the protective roles of autophagy and mitophagy have also been demonstrated for acute myeloid leukemia (AML) cells [10,11]. Mitophagy controls the mitochondrial quality and removes damaged mitochondria through autophagy. Apparently, the mitochondria in CLL cells are prone to accumulate damage and require continual elimination. This phenomenon has been confirmed by the fact that the resistance of CLL cells is associated with mitochondrial reprogramming [4]. Avsec et al. also found that the treatment of CLL cells with autophagy inhibitors significantly disrupted the mitochondrial membrane potential. Nonetheless, a new question arises: can mitophagy inhibitors induce the death of CLL cells and improve the therapeutic outcome of patients with this disease?

Several studies have demonstrated that autophagy-related genes are upregulated in patients with CLL (e.g., *BECN1*, *ATG5*, *GABARAPL2*, *ATG3*, *ATG7*, and *MAP1LC3A*) [12,13,14]. There have been similar observations in other tumors. In one study, there was a high level of autophagy in more than 85% of cases of lung adenocarcinoma. Interestingly, the suppression of basal autophagy via the downregulation of *ATG7* or *ATG13* genes inhibited either the proliferation of lung adenocarcinoma cells or enhanced caspase-dependent and caspase-independent apoptosis, stimulating reactive oxygen species (ROS) formation [15,16]. However, there is no clear data regarding the correlation between the levels of autophagic proteins and a positive or negative prognosis for the patients with blood cancer. This issue requires additional detailed investigation. On the one hand, the knockout of Ulk1 (a very important autophagy player) in mice helped overcome their resistance to venetoclax and impaired leukemic cell homing, delayed disease progression, and improved the survival of mice with AML [17]. In another type of blood cancer—diffuse large B-cell lymphoma—high-level *BECN1* expression is associated with longer survival [18]. Consequently, autophagy does not exert a univocal function as the driver of blood cancers. Notably, only the results of one clinical trial with an autophagy inhibitor for blood cancer have been published. In this trial, hydroxychloroquine and imatinib versus imatinib alone was evaluated in patients with chronic myeloid leukemia (CML) [19]. Importantly, hydroxychloroquine and imatinib provided better results than imatinib alone did. These findings indicate the potential applicability of autophagy inhibitors for the treatment of blood cancer.

Another interesting approach might include combining venetoclax with compounds that influence the NF-κB–p62–NRF2 autophagy-linked axis. Michael Karin’s group detected specific expressions of NRF2 target genes, such as *NQO1*, in CLL cells with the high-level surface representation of the inactive tyrosine protein kinase transmembrane receptor ROR1 [20]. Importantly, high ROR1 levels in patients with CLL correlated with a bad prognosis, including faster disease progression and a shorter overall survival [21]. In cells with high ROR1 levels, p62 accumulation activated NRF2, which induced NQO1 expression. However, the pretreatment of CLL tumor cells with compound 29h, a pro-drug that only becomes active after being metabolized by NQO1, promoted apoptosis and made the cells more susceptible to venetoclax. Moreover, Epstein–Barr virus expression, a predictor of the clinical course and survival of patients with CLL, stabilizes NRF2 in these tumor cells through autophagy inhibition and p62 accumulation [22]. Thus, in virus-associated cancers, the modulation of NRF2 via the NF-kB–p62 pathway or a treatment with NQO1-metabolized pro-drugs may also represent a promising therapeutic approach.

Finally, Avsec et al. investigated whether autophagy inhibitors enhance the rate of venetoclax-induced cell death. The authors reported that these three inhibitors in combination with venetoclax promoted synergic cytotoxicity in CLL cells. Another study examined the autophagy inhibitor VPS34-IN1 and venetoclax and demonstrated significant synergy between these compounds compared with single-agent treatment [14]. These data indicate the protective cellular effect of autophagy in CLL against BH3 mimetics and introduce new strategies to enhance the therapeutic effects of venetoclax on CLL via the inhibition of autophagy pathways.
